# Frequency and spelling of names in the Sierra Leone Ebola Database

**DOI:** 10.11604/pamj.2022.43.141.30408

**Published:** 2022-11-15

**Authors:** Charles Alpren, Lindsay Shively Womack, Frederick Martineau, Elizabeth Kamara, Ansumana Kamara, Amara Jambai, Tushar Singh, Reinhard Kaiser, John Terrell Redd

**Affiliations:** 1 Department of Health and Human Services, Melbourne, Australia,; 2 Centers for Disease Control and Prevention, Atlanta, United States of America,; 3 London School of Hygiene and Tropical Medicine, London, United Kingdom,; 4 Fourah Bay College, University of Sierra Leone, Freetown, Sierra Leone,; 5 African Field Epidemiology Network (AFENET), Freetown, Sierra Leone,; 6 Ministry of Health and Sanitation, Freetown, Sierra Leone,; 7 The Department of Health and Human Services, Office of the Assistant Secretary for Preparedness and Response, Washington DC, United States of America

**Keywords:** Ebola, Sierra Leone, data quality, names, Sierra Leone Ebola Database

## Abstract

Although there is no published analysis of surnames and given names used in Sierra Leone, certain names are common and identical names are frequently encountered. This makes disease tracking and contact tracing difficult. During the Ebola outbreak in 2014-2016, deficiencies in public health information systems in Sierra Leone exacerbated data collection difficulties. The study objective was to examine frequency of names recorded in the Viral Hemorrhagic Fever database (VHF) component of the Sierra Leone Ebola Database (SLED). First names and surnames were standardized by a Sierra Leonean linguist. Frequencies of standardized first names, surnames, full names, and initials were analyzed. The most frequent surname was used by 18.2% of VHF records and the most frequent 20 surnames accounted for 74.1%. The most frequent male first name accounted for 5.5% of VHF records and the most frequent female first name for 4.6%. The 20 most frequent full names accounted for 12.4% of records, and the most frequent initials were used in 7.3% of VHF records. A limited number of names are used in Sierra Leone, which poses a challenge to large public health responses. Algorithms that address inconsistent spelling could be used to improve computer-based databases. Databases must also use variables other than name for identification. The lessons learned in this analysis can assist other investigations, particularly those requiring contact tracing to limit disease spread.

## Essay

In Sierra Leone, certain first names and surnames are common and identical names are frequently encountered. Certain names have multiple spellings and abbreviations, and people might not have a set way of spelling their names. During the Ebola outbreak in 2014-2016, difficulty identifying patients contributed to delays in laboratory results reaching patients, incorrect patients being discharged from Ebola treatment centers, and mistakes made when differentiating patients by initials. Deficiencies in public health information systems exacerbated the data collection difficulties [1,2]. The Sierra Leone Ministry of Health and Sanitation (MoHS) used the Sierra Leone Viral Hemorrhagic Fever (VHF) database as a surveillance system to monitor the Ebola outbreak [3]. The VHF database contains data reported by people (or their relatives) who were suspected of having Ebola disease and collected by case investigators [3]. Although the VHF database is often used for national and international-level analyses, there were considerable difficulties encountered in ensuring consistency and completeness of data when applying it to individual persons [2,4]. For example, some people within the community, especially the elites, may have slightly varied the spelling of their names, such as Fatou instead of the commonly used Fatu, or Ibrahhim instead of Ibrahim. This is to provide them with some uniqueness to commonly used names in the community. A data manager may have changed the spellings to commonly spelled names (e.g. Fatu, Ibrahim). In some cases, records could not be distinguished between individuals with commonly used names (e.g. Mohamed Kamara) and no exact address or unique identification number. If new information (e.g., results of laboratory testing, outcome) became available for the case, data managers might not be able to find a previously created record and had to create a new record. These difficulties impaired patient follow-up and linkage with other databases, such as laboratory and the burial records. These multiple disparate databases have now been combined by MoHS with assistance from the Centers for Disease Control and Prevention to form the Sierra Leone Ebola Database (SLED) [5].

There is no previously published analysis of surnames and given names used in Sierra Leone. We describe the number and commonality of first names, surnames, full names (first name plus surname combination), and initials in the VHF component of the SLED database, and the consistency of spelling of surnames. Understanding the difficulties faced in accurate and consistent recording of names can help in developing ways to improve accuracy of records and simplify linkages across databases. Given the current coronavirus disease pandemic the world is facing, the need to quickly find cases and all contacts highlight the ongoing importance of this work and the need for solutions to limit disease spread. This project was a part of the initial testing of the proposal approval process for SLED data access through the Research Data Center [5,6]. The investigation was approved by CDC in accordance with federal human subject´s protection regulations and CDC policies and procedures as a non-research disease control activity. Review by an institutional review board was not required. The research proposal was prepared by the research team and approved by the MoHS to be conducted by SLED data managers and analyzed by a linguist within Sierra Leone. The Sierra Leonean SLED data team extracted data from the VHF database component of SLED during 2014 to 2015 when all but two cases of Ebola occurred and constructed analytic files. The SLED data team supervisor (AK) and Sierra Leonean linguist (EK) conducted all analyses of extracted files within Sierra Leone to protect MoHS data ownership and data confidentiality. The rest of the research team had access to aggregated data only. Names recorded less than 15 times in the same district of residence were suppressed to maintain confidentiality.

First names and surnames of patients were extracted from SLED in separate files that also contained district of residence and sex. We excluded records that referenced people other than the patient (e.g., baby of) or listed first name or surname as unknown. Prefixes and religious signifiers (e.g., Pa or Chief), middle initials, and middle names were removed. Analysis of first names and surnames was conducted by region (northern, southern, western, and eastern) and nationally. A Sierra Leonean linguist analyzed each record, removing inconsistencies in spelling and typographical errors by assigning standardized first names and standardized surnames to each record. We defined standardized names as a single common spelling given to phonetically identical, phonetically similar, abbreviated, or misspelled names accepted to be the same by the linguist. The frequencies of first name and surname used were obtained by using rates of standardized first names and surnames in VHF records by region of residence weighted by population figures from the 2015 Sierra Leone Census [7] to adjust for differences in region population size. We used a separate file extracted from VHF records to analyze full names. To maintain confidentiality, this file contained the 300 most frequent combinations of standardized first names and standardized surnames with no residence information. From this file, we calculated the proportion of records with each standardized full name. We then analyzed the frequency of sets of initials for each of the 300 most frequent standardized full names. We noted and reported the sex of standardized full names but analyzed male and female together.

From 66,563 records, 1,350 differently spelled first names were recoded into 263 standardized first names. Adjusted for region population size, the most frequent male standardized first name, Mohamed, accounted for 5.5% of VHF records and the most frequent female standardized first name, Fatmata, accounted for 4.6%. The 20 most frequent standardized first names accounted for 46.5%, and the 50 most frequent standardized first names accounted for 69.5% of VHF records. There were 33 standardized first names in the 20 most frequent first names in all 4 regions (Table 1). From 87,760 records, 1,080 differently spelled surnames were recoded into 366 standardized surnames. Adjusted for region population size, the most frequent standardized surname, Kamara, accounted for 18.2% of VHF records. The 20 most frequent standardized surnames accounted for 74.1%, and the 50 most frequent accounted for 85.5% of VHF records. There was some variation in surnames across the regions of Sierra Leone, with 31 standardized surnames accounting for the 20 most frequent names in all 4 provinces (Table 1).

**Table 1:** prevalence of the 20 most frequent standardized first names and standardized surnames in the viral hemorrhagic fever database by region adjusted for regional population size - Sierra Leone, 2014-2015

	Whole country		Western region (Western Area Urban, and Western Area Rural)		Northern region (Port Loko, Kambia, Koinadugu, Tonkolili, and Bombali)		Eastern region (Kono, Kenema, and Kailahun)		Southern region (Bo, Bonthe, Moyamba, and Pujehun)	
Rank	First name (%)	Surname (%)	First name (%)	Surname (%)	First name (%)	Surname (%)	First name (%)	Surname (%)	First name (%)	Surname (%)
1	Mohamed (5.5)	Kamara (19.1)	Mohamed (5.5)	Kamara (19.4)	Mohamed (5.9)	Kamara (21.0)	Fatmata (4.9)	Kamara (13.6)	Mohamed (5.2)	Kamara (17.5)
2	Fatmata (4.6)	Sesay (8.7)	Fatmata (5.4)	Koroma (10.2)	Kadiatu (4.2)	Bangura (10.2)	Mohamed (4.6)	Sesay (7.4)	Fatmata (4.7)	Koroma (8.3)
3	Kadiatu (3.7)	Bangura (8.5)	Kadiatu (4.0)	Sesay (9.0)	Fatmata (3.9)	Sesay (9.4)	Ibrahim (2.8)	Kargbo (6.4)	Abdulai (3.2)	Sesay (6.4)
4	Abdulai (3.2)	Koroma (8.3)	Abdulai (3.7)	Bangura (8.9)	Aminata (3.5)	Conteh (7.5)	Kadiatu (2.5)	Koroma (6.1)	Kadiatu (3.2)	Bangura (5.5)
5	Aminata (3.1)	Conteh (6.2)	Aminata (3.5)	Conteh (6.2)	Mariatu (3.4)	Koroma (6.8)	Abdulai (2.5)	Conteh (3.7)	Borbor (2.9)	Kargbo (5.0)
6	Ibrahim (3.0)	Kargbo (5.3)	Isatu (3.3)	Mansaray (6.0)	Ibrahim (3.2)	Turay (5.9)	Aminata (2.4)	Bangura (3.6)	Ibrahim (2.2)	Conteh (4.5)
7	Mariatu (2.6)	Mansaray (4.1)	Ibrahim (3.2)	Kargbo (5.0)	Abdulai (3.2)	Kargbo (5.3)	Sahr (2.2)	Mansaray (2.9)	Adama (2.1)	Mansaray (2.9)
8	Adama (2.5)	Turay (3.8)	Mariatu (2.9)	Kanu (3.0)	Ya (3.2)	Kanu (4.8)	Hawanatu (2.2)	Jalloh (2.6)	Aminata (2.1)	Kanu (2.0)
9	Ya (2.4)	Kanu (3.4)	Adama (2.9)	Turay (2.8)	Adama (2.7)	Mansaray (2.7)	Borbor (2.1)	Turay (2.2)	Mamie (2.0)	Fofanah (2.0)
10	Isatu (2.2)	Jalloh (2.4)	Abubakarr (2.5)	Jalloh (2.8)	Isatu (2.2)	Sankoh (2.5)	Tamba (1.7)	Kallon (2.2)	Mariama (1.8)	Jalloh (1.8)
11	Hawanatu (1.8)	Sankoh (1.5)	Ya (2.4)	Dumbuya (1.2)	Osman (1.8)	Jalloh (1.9)	Mariama (1.7)	Tarawalie (2.1)	Abu (1.8)	Turay (1.7)
12	Osman (1.5)	Fofanah (1.2)	Hawanatu (2.0)	Fofanah (1.1)	Hawanatu(1.7)	Fornah (1.2)	Abu (1.7)	Fofanah (1.9)	Ya (1.4)	Bah (1.7)
13	Mabinty (1.4)	Bah (1.1)	Osman (1.7)	Bah (1.1)	Mabinty (1.7)	Dumbuya (1.0)	Adama (1.6)	Musa (1.8)	Isata (1.4)	Fornah (1.4)
14	Hassan (1.4)	Dumbuya (1.1)	Hassan (1.6)	Sankoh (1.1)	Hassan (1.6)	Bah (0.9)	Isata (1.5)	Bockarie (1.5)	Hawa (1.4)	Sheriff (1.2)
15	Mariama (1.4)	Fornah (0.9)	Mabinty (1.6)	Barrie (0.9)	Foday (1.5)	Fofanah (0.9)	Musa (1.4)	Bah (1.5)	Salamatu (1.3)	Massaquoi (1.1)
16	Foday (1.3)	Kabia (0.8)	Alie (1.4)	Kabia (0.9)	Alie (1.4)	Kabia (0.9)	Sia (1.4)	Kanu (1.4)	Foday (1.3)	Musa (1.0)
17	Borbor (1.3)	Barrie (0.8)	Mariama (1.3)	Tarawalie (0.7)	Mariama (1.3)	Samura (0.9)	Mariatu (1.4)	Sheriff (1.4)	Musa (1.2)	Dumbuya (0.9)
18	Alie (1.2)	Tarawalie (0.7)	Foday (1.2)	Kabba (0.7)	Salamatu (1.3)	Barrie (0.8)	Foday (1.3)	Momoh (1.4)	Joseph (1.2)	Lahai (0.9)
19	Salamatu (1.2)	Kallon (0.6)	Alpha (1.2)	Samura (0.7)	Alimamy (1.2)	Tholley (0.7)	Kumba (1.2)	Fornah (1.0)	Marie (1.1)	Kallon (0.9)
20	Abubakarr (1.2)	Samura (0.6)	Salamatu (1.2)	Abdulai (0.6)	Abu (1.2)	Fullah (0.5)	Mamie (1.2)	Kanneh (1.0)	Isatu (1.1)	Kanneh (0.8)

The 20 most frequent standardized full names accounted for 12.4% of VHF records, and the 300 most frequent accounted for 49.2%. The most frequent name (a male name) was used in 1.2% of VHF records. The next 2 most frequent names (both female names) were used in 1.1% and 0.82% of VHF records, respectively. In the 20 most frequent names, there were 12 sets of initials. The most frequent initial set, AK, was used in 7.3% of VHF records and the second most frequent, MK, was used in 6.1% of records. The 20 most frequent sets of initials accounted for 39.1% of records in the VHF database. Although the scope of this paper does not include a full analysis of spelling inconsistency, we noted transcription errors, omitted letters, and inserted letters. The 20 most frequent standardized surnames were spelled 368 different ways in VHF records. We observed multiple instances of missing last letter (e.g., Kamar instead of Kamara) or an extra last letter (e.g., Kamarah instead of Kamara). Also, in some records, the VHF field for the surname contained a first name and vice versa.

These results illustrate the difficulty of record keeping in Sierra Leone during the Ebola outbreak. A relatively limited number of names are used in Sierra Leone; the most frequent names have multiple spellings within VHF records, which appears attributable to both real variation and recording error. Initials are shared between many of the most frequent full names. A limited number of names are used by a substantial proportion of the population, which contrasts with the United States where the most frequent surname (Smith) is used by 0.85% of the population, and the most frequent full name is used by 0.016% [8]. Fixed inherited surnames are far from ubiquitous around the world and can be a legacy of colonial and authoritarian control through taxation and conscription [9], including in Sierra Leone [10], which may continue to influence their access and uptake. Secondary identifiers such as middle initials and precise address, are useful but can be difficult to apply with operational challenges during fieldwork. In communications outside the direct clinical setting, such as when talking about infection clusters, it is common to use patients´ initials. However, this causes confusion in an environment in which only first name and surname are reliably recorded, and where two initials, AK and MK, accounted for approximately 14% of the patient population. Spelling in VHF records was dependent on both the recorder and reporter. Response personnel often copied case identification forms and transcribed forms into the VHF database from memory because of operational challenges in the field [2]. Differences in Sierra Leonean local languages between reporter and recorder and low adult literacy in Sierra Leone [11] may have resulted in spelling inconsistencies. Inconsistent spelling caused difficulty in linking records during the outbreak, and in linking databases during the development of SLED.

This study has limitations both because of the standardization process and the analysis of the standardized names. The standardization process for this study was subjective and decided at the discretion of the Sierra Leonean linguist. Distinguishing between true variants and recording errors was difficult and often debated among the research team. Different tribes spell and pronounce names differently, so our analysis maintained tribal variations in spelling and did not standardize them. Other standardizations removed abbreviated names that are not universally applied. For example, Abdulai is often shortened to Abdul. Both names were assigned the standardized name, Abdulai. Although the standardization process removed common age and religious monikers such as Pa, the name Ya remained for analysis. This name is used as both a stand-alone name and a prefix denoting an elderly woman added to signify respect. This analysis combined prefixed names with their unprefixed counterparts, for example, combining Yasatu and Isatu as into a single standardized first name, and treated Ya when found alone as a separate standardized first name.

We did not analyze the variation or recording of middle initials and middle names, which are commonly used for identification in Sierra Leone. Published data concerning use of middle names or initials to help with identification during the Ebola outbreak are not available, but the authors´ experiences were that they were not frequently used within the Ebola response to help identify patients. The VHF database contains records of people exhibiting symptoms of Ebola and may not be representative of the Sierra Leonean population. Further, the database contains a disproportionate number of records from western and northern regions, which had higher numbers of Ebola cases and were affected later in the outbreak [12] when the response mechanism, including recording in VHF database was more established [13]. Because names often correspond to ethnicity, exhibiting regional variation in their frequency of use, this study may have underestimated the frequency of names common in eastern and southern regions. To maintain confidentiality during analysis we looked at full names and initials for the 300 most common standardized full names only. These names accounted for 49% of the VHF database, but we note that many of the names not in the 300 most common would have had the same initials as the commoner names. Hence, our analysis underestimated prevalence of common initials.

Finally, incorrect assignment of name fields could not be differentiated from names normally used as first names being used as surnames. Although in Sierra Leone people inherit their father´s surname, people also sometimes take the first name of their father as their surname. A limited number of names are commonly used in Sierra Leone, which makes identifying a specific person difficult. Furthermore, during the Ebola outbreak, inconsistent spelling within records, low age data quality in adult patients [14], and discrepancy in reporting home addresses were common. These factors, and the absence of a personal unique identifier, such as social security number, contributed to mistakes and inefficiencies during the Ebola response, including delays in laboratory results reaching patients and incorrect identification of patients. Standardizing names within databases would not have helped appreciably with identification difficulties faced in the field during the Ebola outbreak, because of people having similar names. Furthermore, standardizing names would result in a loss of name diversity, which might otherwise aid identification and cross-linking if spelling variations were recorded consistently. Algorithms that address inconsistent spelling could be used in the background processes of computer-based databases to allow easier linkage across databases. However, to maximize usefulness of a database in the field, accurate identification and differentiation of people recorded within it are vital. Where variation among names is low or accuracy of spelling difficult to achieve, databases must use other techniques for patient identification. These could include using unique text-based, numerical, or other identifiers such as bar codes, whether preassigned or generated for the response. In addition, responders must emphasize the importance of accurate recording of information such as middle names, address, and next of kin from the time of initial patient contact. Though this work represents findings from Sierra Leone, it is likely that many other countries and regions have similar name constraints. Ensuring that quality descriptive data is collected especially for diseases where rapid testing is not available for patients and providers to receive results before returning home and for diseases requiring contact tracing to minimize disease spread.

**Disclaimer:** the findings and conclusions in this report are those of the authors and do not necessarily represent the official position of the Centers for Disease Control and Prevention or other organizations.
